# Difficult Mask Ventilation in Penetrating Facial Trauma Due to Animal Attack: A Unique Challenge in the Emergency Department

**DOI:** 10.7759/cureus.23831

**Published:** 2022-04-04

**Authors:** Himanshi Baid, Poonam Arora, Rajnish K Arora, Hannah Chawang, Aadya Pillai

**Affiliations:** 1 Emergency Medicine, All India Institute of Medical Sciences, Rishikesh, IND; 2 Neurosurgery, All India Institute of Medical Sciences, Rishikesh, IND

**Keywords:** cannot intubate cannot ventilate, first pass success, airway management, airway assessment, bear bite, penetrating facial trauma, difficult airway, difficult mask ventilation

## Abstract

Penetrating facial trauma can be a life-threatening condition, especially due to its impact on the airway. In a facial trauma, there is a distortion in the basic anatomy of the affected, making it a particularly difficult situation for managing the airway. Challenging intubation scenarios have been widely explored in the literature; however, difficult to ventilate situations have been undermined. We describe a case of a 35-year-old female who presented with a history of animal attack on the face. The extent of penetrating facial trauma warranted the need to secure the airway. Preserving spontaneous breathing and using an oral endotracheal tube for oxygenation saved the airway manager from cannot intubate and cannot oxygenate situation in a facial trauma patient.

Difficult to mask ventilate while arranging for a definitive airway can be more pressing and challenging for the emergency physician. It also jeopardizes the patient's life, whose survival may only depend on acquiring the patency of the airway. Facial trauma patients may be conscious and spontaneously breathing, leading to the missed or delayed intervention in the airway; hence, prompt assessment and management of the airway in all facial trauma are of utmost importance.

## Introduction

Airway management is the first and most crucial aspect of patient management in most emergencies. It is of prime importance for emergency physicians to effectively manage and promptly identify difficult airway situations. This allows for more efficient management in terms of equipment, personnel, and team dynamics. Difficult airway management generally includes difficult laryngoscopy, difficult intubation, and sometimes difficult mask ventilation. An incidence of one in 690 impossible to ventilate situations has been reported making it difficult to ventilate in a very infrequent yet challenging situation [1].

Maxillofacial trauma is an important life-threatening situation requiring prompt identification of threatened airway and effectively managing the same, fighting the odds of distorted anatomy and constraints of time. The incidence of difficult airway in patients with maxillofacial trauma was seen in 20% of cases in the Indian setting [2]. There is, however, no proper evidence on difficult to ventilate situations.

We discuss a case of an animal attack (bear bite) causing penetrating facial trauma, which posed a unique challenge for airway assessment and management in the emergency department of a tertiary care center at the foothills of the Himalayas.

## Case presentation

**Figure 1:**
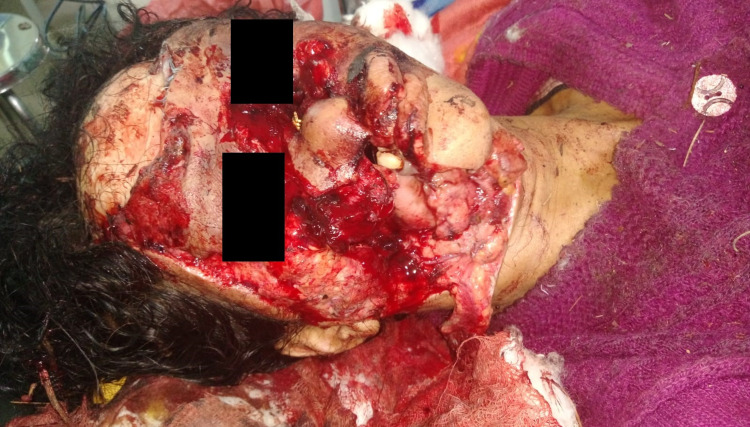
Penetrating facial injury of the patient.

We received a 35-year-old female in the emergency department with a history of animal attack (bear bite) about two hours prior to her presentation at the emergency department. She had sustained penetrating trauma to the face (Figure 1). The patient was evaluated as per the Advanced Trauma Life Support (ATLS) protocol, and the extent of facial injury warranted a need to secure the airway.

Anatomical assessment of the airway is commonly assessed using the LEMON (Look, Evaluate the 3-3-2 rule, Mallampati score, Obstruction, and Neck mobility) score in our setup. However, this patient’s facial trauma led to distorted anatomy, increasing airway obstruction potential. High suspicion of aspiration was kept as the patient was disoriented (Glasgow Coma Scale score: E3V1M5). Due to the pain, she did not allow assessment of mouth opening and Mallampati score. The lacerated nature of the injury caused a loss of supporting structures of the oral airway and tone of tongue muscles. The patient’s tongue falling backward caused snoring. There was profuse bleeding from the oral and nasal cavity. The LEMON scoring could not be of much use here, as most criteria could not be assessed properly. However, the overall clinical assessment of the patient revealed multiple red flags for preparing to secure the airway. Airway assessment by suboptimal mask seal (M), obstruction or obesity (O), advanced age (A), no teeth (N), and stiffness of lungs (S) or MOANS showed a situation of difficult mask ventilation (DMV); however, the ease of intubation was at best assessed to be dubious. Assessment of breathing parameters was within normal limits with no evidence of crepitations on lung auscultation, despite the distorted airway. The physiological and biochemical parameters of the patient revealed tachycardia (heart rate of 120/min), hypotension (88/56 mmHg), and mild acidosis. Two large-bore intravenous cannulas (16 gauge each) were secured and 500 ml Ringer lactate was given in bolus over 15 minutes for the same.

Due to irregularities seen in the airway assessment, the patient’s airway was considered a difficult airway. Endotracheal intubation was kept as plan A for securing the airway, the supraglottic airway was kept as plan B, and needle cricothyroidotomy as plan C, with a definite plan of tracheostomy later on. Due to extensive facial injuries, an ENT surgeon was kept on standby in emergency need of a surgical airway in the form of tracheostomy.

**Figure 2:**
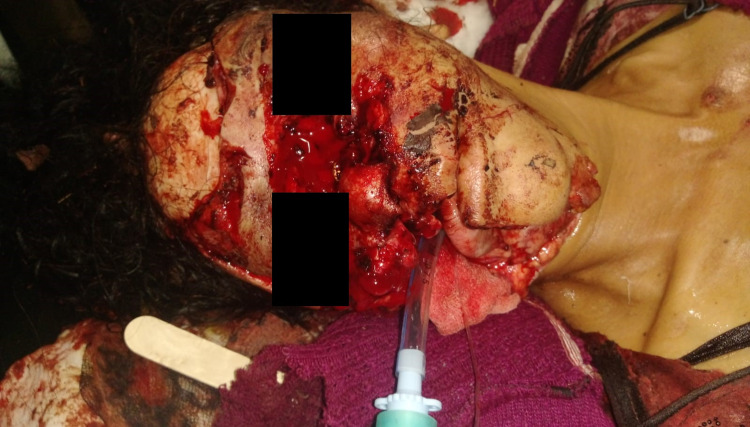
The patient after establishing a definitive airway.

In view of hypotension (initial heart rate: 120/min; BP: 88/56 mmHg), IV ketamine 1 mg/kg was used to induce the patient after premedication with glycopyrrolate (40 mcg/kg IV). Pre- and para-oxygenation were done with a 7.5 mm endotracheal tube placed past the tongue, connected to the Bain’s circuit to circumvent the difficulty to ventilate the patient. With the patient breathing spontaneously after induction with ketamine, laryngoscopy was performed with Macintosh No. 4 blade after thorough suctioning of the oral cavity; the same endotracheal tube was advanced through the vocal cords after application of cricoid pressure, without using any muscle relaxant (Figure 2). Tear at the right angle of the mouth was used as an advantage in this patient as it provided room to manipulate the laryngoscope and endotracheal tube. After emergency department stabilization, the patient was shifted to the operating room for control of bleeding and debridement of the injury by the trauma and plastic surgery team. Tracheostomy was undertaken under sedation and analgesia in the operating room. As the patient was breathing spontaneously, the patient was kept on T piece with adequate sedation and shifted to the ICU for further stabilization after some time. The patient did not require mechanical ventilation during her hospital stay and gradually her oxygen requirement was tapered with the tracheostomy tube in situ. The patient required multiple surgical interventions by the plastic surgery team, which was followed by flap repair of the wound after achieving a healthy tissue bed during her hospital stay of 45 days. The patient was then discharged with advice to follow up and tracheostomy in situ and a plan for gradual decannulation.

## Discussion

According to the ATLS approach, airway loss is a more potent killer than breathing or circulatory problems. Thus, any life-saving intervention should be initiated with airway management, wherever required [3]. The problems encountered during airway management can lead to a graver outcome in trauma patients [2-5]. Each facial trauma is a unique challenge in the aspect of airway management. The distortion of anatomy makes every facial trauma a difficult airway situation either in terms of difficult to ventilate or difficult to intubate, or even both at times [2,5,6].

The choice of approach is based on the patient’s ability to maintain a patent airway and their oxygenation status. Our patient was altered, unable to hold secretions but breathing spontaneously and maintaining adequate oxygenation. Assessment of the airway showed difficult mask ventilation and a plan was made to secure the airway while preserving spontaneous respiration. The use of an oral endotracheal tube past the tongue bypassed upper airway obstruction and provided a visible guide of the adequacy of spontaneous breathing from bag movements and square waveform of end-tidal carbon dioxide. Securing the airway in the emergency itself allows easy transition to a definitive long-term airway in facial trauma patients.

Distortion of facial structures may make obtaining a seal with a mask difficult and positive pressure ventilation may result in aspiration of blood. Providing oxygen with nasopharyngeal airway (NPA) is an option in patients with difficult mask ventilation, but may not be possible in all facial trauma patients. Some facial trauma patients may even be easier to intubate due to lack of any mobility restriction by soft tissues. All of the above parameters were seen in our patient, making the management be tailor cut individually for our patient.

Facial trauma patients are prone to the development of aspiration and hypoxemia, which needs to be a priority while managing these patients [5]. Adequate positioning and suction-assisted laryngoscopy assisted decontamination (SALAD) technique can prevent aspiration [7]. Facial injuries are associated with airway contamination with blood, which limits the success of first intubation, especially with devices such as a fibreoptic endoscope and video-laryngoscope [8]. Moreover, blood in the airway can lead to several complications related to difficult airway management and/or aspiration. Altered sensorium, loss or reduction in protective airway reflexes, delayed gastric emptying, and full stomach further add to the risk of vomiting and aspiration during airway management [5,6]. Our patient was altered and was comfortable only in the upright position because of the trauma site. This position had the advantage of easy drainage of blood and better functional residual capacity (FRC). One rigid suction was placed in the upper esophagus (immediately after induction of the patient) and the second suction was used during laryngoscopy while managing the patient to reduce the risks of aspiration [7].

Preoxygenation with bag-mask ventilation (BMV) in patients with facial trauma may be difficult, as also reoxygenation with mask ventilation during rapid sequence intubation (RSI) in case of an unsuccessful first attempt. Oxygenation was provided by keeping the endotracheal tube in the oropharynx, as creating an adequate seal of BMV was not possible due to the lack of facial structures in our patient.

## Conclusions

Airway management is the most crucial aspect of managing a facial trauma patient in the emergency department. The prompt evaluation of the airway and anticipation of the possible difficulties encountered during its management not only helps in adequate preparation but may also improve the patient outcome, as was seen in our case. The commonly used scores for airway assessment may not be able to give a clear picture in patients with facial trauma due to distortion in anatomy and the possibility of anticipated airway obstruction (in the form of blood, secretions, or foreign bodies). Hence, each case has to be assessed and managed individually.
